# An integrated prognostic model incorporating clinical characteristics, multiparametric MRI radiomics, deep learning features, and intratumoral heterogeneity for glioma patients

**DOI:** 10.3389/fonc.2026.1873715

**Published:** 2026-09-21

**Authors:** Yanru Yang, Keqi Su, Kaiqiang Tang, Kang Li

**Affiliations:** 1 School of Imaging Medicine, North Sichuan Medical College, Nanchong, Sichuan, China; 2 Department of Radiology, Chongqing Academy of Medical Sciences, Chongqing General Hospital, Chongqing University, Chongqing, China

**Keywords:** deep learning, glioma, intratumoral heterogeneity, multiparametric MRI, prognostic prediction, radiomics

## Abstract

**Introduction:**

Gliomas are the most common primary malignant tumors of the central nervous system, characterized by significant biological heterogeneity and poor prognosis. Multiparametric MRI-based image analysis has shown promise in prognostic assessment; however, effective integration of deep learning (DL), radiomics, and intratumoral heterogeneity (ITH) quantification remains underexplored. This study aims to develop an integrated prognostic model incorporating clinical and multiparametric MRI-derived features and systematically evaluate the predictive performance of different feature combinations.

**Methods:**

This retrospective, multicenter study included 183 patients from the UCSF-TCIA cohort as the training set and 109 patients from Chongqing General Hospital as an independent test set. Based on preoperative multiparametric MRI—including T1-weighted imaging (T1WI), T1-weighted contrast-enhanced imaging (T1CE), T2-weighted imaging (T2WI), T2 fluid-attenuated inversion recovery (T2-FLAIR), diffusion-weighted imaging (DWI), and apparent diffusion coefficient (ADC)—radiomics, DL, and 2D/3D ITH features were extracted. Feature selection was performed using Least Absolute Shrinkage and Selection Operator–Cox (LASSO-Cox) regression, and both single and fusion models were constructed. Feature-level integration was achieved using a two-stage LASSO-Cox regression to leverage the complementary nature of information from different sources fully. Performance was quantified using the Harrell concordance index (C-index) and validated via Kaplan–Meier survival analysis, time-dependent receiver operating characteristic (ROC) analysis, calibration curves, and decision curve analysis (DCA).

**Results:**

The fusion model integrating DL, radiomics, ITH, and clinical features (DenseNet121_DL+Rad+ITH) achieved C-indices of 0.801 (95% CI: 0.750–0.853) and 0.710 (95% CI: 0.638–0.783) on the training and test sets, respectively, outperforming all single models. The corresponding stacking ensemble yielded a comparable test C-index of 0.701. Kaplan–Meier analysis confirmed significant risk stratification (P < 0.001). Time-dependent ROC analysis showed AUCs of 0.705, 0.721, 0.746, 0.800, and 0.873 at 12, 18, 24, 30, and 36 months. Calibration curves demonstrated good agreement between predicted and observed survival probabilities. DCA indicated favorable clinical net benefit for medium- to long-term survival prediction (24–36 months).

**Conclusions:**

An integrated prognostic model combining clinical characteristics with multiparametric MRI-derived features (DL, radiomics, and ITH) demonstrates strong prognostic potential for glioma patients. Fusion strategies generally outperform single-feature models; however, incremental gains from fusion are modest, and prospective validation in larger cohorts remains necessary.

## 1 Introduction

Gliomas are the most common primary malignant tumors of the central nervous system (CNS), with a median survival time of less than 21 months (1, 2). These tumors exhibit significant biological heterogeneity, ranging from low-grade astrocytomas to highly malignant glioblastomas. Glioblastomas, in particular, are characterized by high invasiveness, rapid progression, a tendency to develop treatment resistance, and an extremely poor prognosis (3). Despite continuous advances in comprehensive treatment strategies—including surgery, radiation therapy, and chemotherapy—survival benefits for patients with high-grade gliomas remain very limited (4). The infiltrative growth pattern of gliomas, their complex intratumoral structural variability, and high tendency to recur significantly limit treatment efficacy (5). Therefore, accurate assessment of patient survival outcomes is crucial for developing personalized treatment strategies, optimizing clinical decision-making, and improving the efficiency of follow-up management (6, 7).

Currently, the prognosis of gliomas is primarily assessed based on histological classification, molecular markers, and imaging characteristics. Accurate prediction of overall survival (OS) is of central importance in optimizing patient management, early identification of high-risk cases, and personalized treatment planning (8).At the same time, factors such as age, WHO histological grade, isocitrate dehydrogenase (IDH) mutation status, and gender have been widely shown to be closely associated with the prognosis of glioma patients (9). However, prognostic models that rely solely on clinical and pathological variables often fail to fully capture information on intratumoral phenotypic heterogeneity (10). Therefore, multidimensional integration of clinical and imaging features is expected to complement each other, thereby improving the accuracy and clinical utility of prognostic predictions (11). Furthermore, magnetic resonance imaging (MRI) can noninvasively and multidimensionally characterize features such as tumor volume, necrosis, blood supply, and edema, T2 fluid-attenuated inversion recovery (T2-FLAIR) is highly sensitive to peritumoral edema and non-enhancing infiltration; T1-weighted contrast-enhanced (T1CE) imaging reveals blood-brain barrier disruption and active lesions; T2-weighted imaging (T2WI) identifies intratumoral fluid and areas of necrosis; and diffusion-weighted imaging (DWI) and apparent diffusion coefficient (ADC) quantify tumor cell density. Compared to a single sequence, integrating the information from the multiple sequences mentioned above provides a more comprehensive characterization of the biological characteristics of tumors and offers richer input for prognostic modeling (12–14). Radiomics systematically extracts quantitative features from images using predefined mathematical operators, providing an objective reflection of tumor microstructural information that is difficult to detect with the naked eye. Deep learning (DL), on the other hand, utilizes convolutional neural networks to automatically learn multi-level abstract representations from raw images, enabling the capture of higher-order visual patterns that are difficult to quantify using traditional radiomics, such as edge geometry, texture hierarchy, and spatial contextual information (15, 16); Compared with radiomics features, DL features are generated in a data-driven manner, and the two are inherently complementary in terms of information dimensions (17). Consequently, the integration of DL and radiomics has become a hot research topic in recent years, providing a new technical approach for mining information from multiparametric MRI and demonstrating great potential in prognostic prediction (18–21).

It is worth noting that radiomic features are inherently based on global statistical distributions and lack a direct representation of the spatial organization of different phenotypic regions within a tumor. However, the intratumoral heterogeneity (ITH) score recently proposed by Li et al. (22) offers a new approach to addressing this issue. By quantifying the spatial connectivity and dispersion of different phenotypic subregions, this method characterizes the structural complexity of tumors from a spatial-topological perspective, constituting a measure of spatial heterogeneity independent of traditional radiomics. ITH places greater emphasis on the spatial organization of different phenotypic regions and holds potential value in assessing tumor aggressiveness and prognosis (23, 24). Previous studies have primarily focused on solid tumors such as lung cancer and breast cancer (25–27), while research on brain tumors remains limited. Furthermore, existing studies are mostly based on analysis of the two-dimensional (2D) maximum cross-section, making it difficult to fully reflect the tumor’s heterogeneity at the volumetric level. To address this, Zuo et al. (28) proposed a three-dimensional (3D) ITH scoring method capable of modeling across the entire valid range, which is expected to provide more comprehensive structural information. Therefore, the quantitative characterization of tumor heterogeneity holds promise as a complementary approach in glioblastoma prognosis research. Based on this, the present study combined clinical characteristics, multiparametric MRI, DL, and radiomics with 2D and 3D ITH score features to develop a survival prediction model.

## 2 Methods

### 2.1 Data source

This study included a training set of 183 patients and a test set of 109 patients. The training set was derived from the UCSF-PDGM dataset (29, 30) in the Cancer Imaging Archive (TCIA), which was constructed by the University of California, San Francisco (UCSF). Serving as the standardized data source for this study, this dataset contains preoperative multi-sequence MRI data and clinical information for patients with pathologically confirmed diffuse gliomas between 2015 and 2021. For the test set, we included patients treated at Chongqing General Hospital between January 2020 and July 2025 whose brain gliomas had been pathologically confirmed via surgical resection specimens. All cases included diffuse gliomas classified as World Health Organization (WHO) CNS grades 1–4; among these, WHO grades 1–2 are defined as low-grade gliomas, and WHO grades 3–4 are defined as high-grade gliomas.

Inclusion criteria primarily included (1): Complete preoperative MRI images, including T1WI, T1CE, T2WI, T2-FLAIR, and DWI (including ADC maps), as well as a complete pathology report and follow-up information (2); Preoperative cranial MRI showing a well-defined lesion without significant artifacts. Exclusion criteria (1): Recurrence, chemoradiotherapy, or other antitumor treatments (n=9) (2); Poor MRI image quality or significant artifacts rendering the images unusable for analysis (n=13) (3); Concurrent intracerebral diseases such as cerebral hemorrhage or cerebral infarction (n=3) (4); History of other malignant tumors (n=2).

This study is a retrospective, multicenter study that was approved by the Ethics Committee of Chongqing General Hospital, with the informed consent requirement waived; the use of data from public databases was also exempt from the consent requirement. Demographic and clinical data were derived from medical records. Clinical variables collected included age, sex, WHO grade, isocitrate, dehydrogenase (IDH) mutation status, extent of resection (EOR), and postoperative adjuvant therapy. The primary endpoint was overall survival (OS), defined as the time from the date of surgery to all-cause death. No patients were lost to follow-up during the study period; those still alive as of the study cutoff date were recorded as such.

### 2.2 MRI acquisition and image preprocessing

All preoperative MRI scans in the UCSF-PDGM dataset were performed using a 3.0 T magnetic resonance imaging system (Discovery 750, GE Healthcare) with an 8-channel dedicated head coil. Examinations for patients at our hospital were performed using either the United Imaging uMR 670 1.5 T or Skyra MR E11 3.0 T MRI scanners. The scanning sequences included T1WI, T1CE, T2WI, T2-FLAIR, and DWI, and cranial MRI data were acquired using a 20-channel phased-array head coil. The DWI sequence used a B-value of 1000 s/mm² with diffusion encoding in six orthogonal directions; ADC maps were generated from the DWI images using diffusion gradient encoding. All of the above images served as multichannel inputs for the model; detailed acquisition parameters for the relevant sequences are provided in **Supplementary Table 1**.

During the multi-center data integration process, all scan images were processed according to a standardized preprocessing workflow: First, the N4 algorithm was used for bias field correction to reduce signal inhomogeneity and make the images more balanced overall; second, voxel values were standardized to a distribution with a mean of zero and a variance of one, thereby ensuring comparability among different images; third, all images were uniformly resampled to 1×1×1 mm³ isotropic voxels. Based on this, using ANTs (version 3.16) and a mutual information metric, all sequences are aligned to a unified coordinate system via rigid transformations to ensure spatial consistency of all information. The entire processing workflow preserves the integrity of the original anatomical structures while supporting subsequent detailed segmentation and image feature extraction. The overall study design workflow is shown in **Figure 1**.

**Figure 1:**
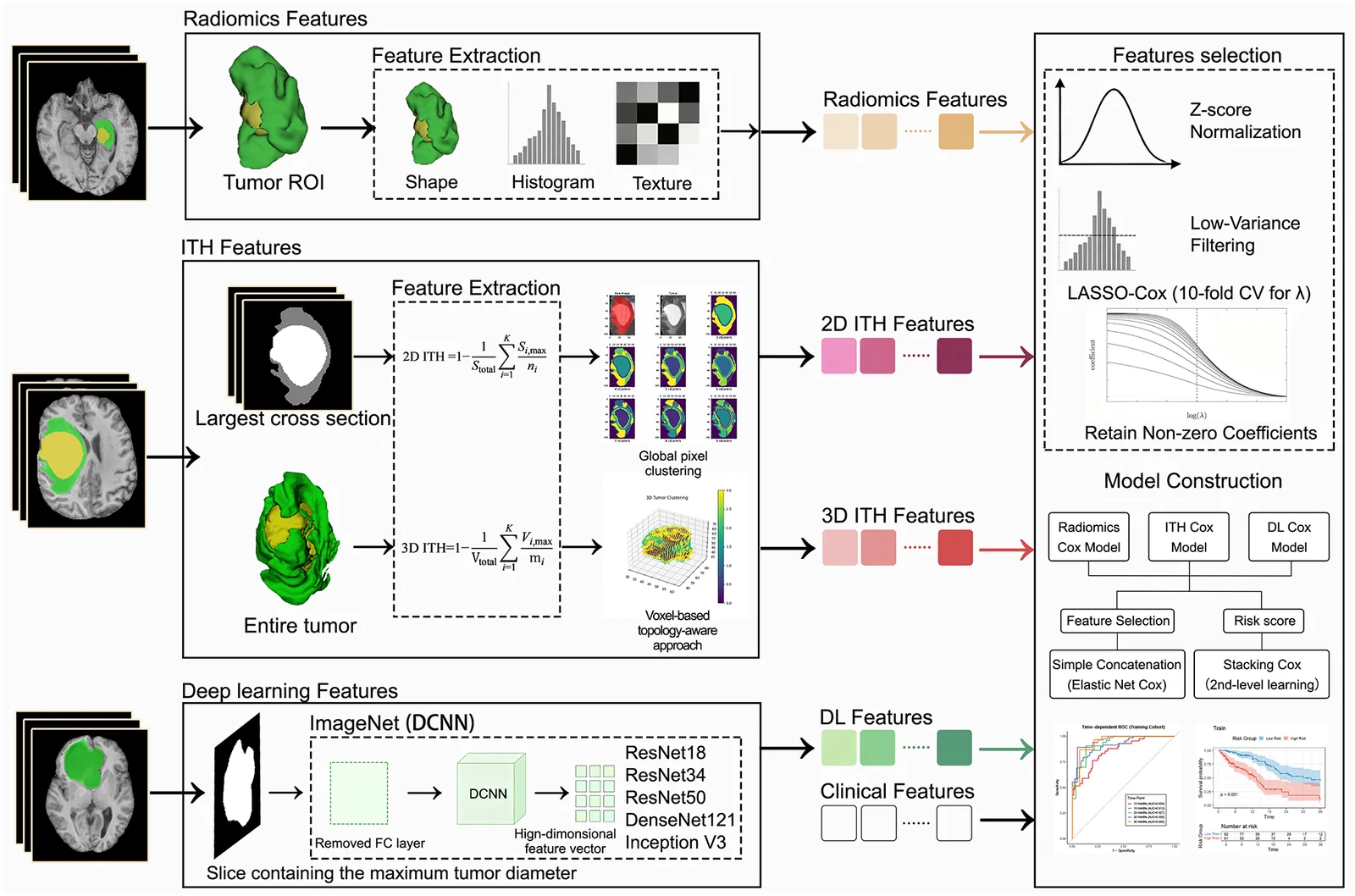
The workflow of this study.

### 2.3 Radiomics feature extraction

Based on preprocessed and registered multiparametric MRI images, this study clearly defined two regions of interest (ROIs) for manual segmentation: the whole tumor (WT) and the peritumoral edema (PE). The WT encompasses the contrast-enhancing tumor core, non-enhancing tumor components, and necrotic areas. It was delineated primarily based on abnormal signals observed in T2WI and T2-FLAIR sequences, with T1CE sequences used to confirm its enhancement boundaries. The PE, on the other hand, is defined as the set difference between the high-signal areas on T2-FLAIR outside the tumor margin and the WT region, to assess tumor infiltration and characteristics of the peritumoral microenvironment. The aforementioned segmentation process relied primarily on T2-FLAIR sequences, with T1CE sequences used for reference and comparison. Regions of interest (ROIs) for all lesions were manually delineated on a slice-by-slice basis by two physicians with extensive experience in neuroimaging (one attending radiologist with 8 years of experience and one chief physician with 25 years of experience) using ITK-SNAP (version 3.8.0). To ensure the accuracy of the segmentation, all initial segmentations were reviewed and corrected by the chief physician to reach a final consensus. To assess the reproducibility of radiological features, data from 50 randomly selected patients were used; inter-observer agreement was evaluated using the intraclass correlation coefficient (ICC). Only features with an ICC greater than 0.75 were retained for further analysis. Radiomic feature extraction was performed using PyRadiomics in a Python environment. The following feature families were extracted for each image type: 14 shape features, 18 first-order statistical features, 24 gray-level co-occurrence matrices (GLCM), 16 gray-level run-length matrices (GLRLM), 16 gray-level size matrices (GLSZM), 14 gray-level dependency matrices (GLDM), and 5 neighborhood gray-level difference matrices (NGTDM). A total of 1,130 radiomic features were extracted from each MRI sequence.

### 2.4 Deep learning feature extraction

This study introduces several deep convolutional neural networks (DCNNs) pre-trained on ImageNet, including ResNet18, ResNet34, ResNet50, DenseNet121, and Inception V3, which are primarily used for deep feature extraction from tumor images; We uniformly removed the original fully connected classification layers from each network and used the output of the Global Average Pooling (GAP) layer as the deep feature extraction node (31, 32), thereby obtaining feature vectors that possess global semantic representation capabilities. The feature dimensions of each network are as follows: ResNet18 (512-dimensional), ResNet34 (512-dimensional), ResNet50 (2048-dimensional), DenseNet121 (1024-dimensional), and Inception V3 (2048-dimensional); the five network architectures were each retained as independent feature matrices. Regarding image processing, for each patient, a representative slice corresponding to the tumor’s maximum diameter was selected as the basis for analysis (33–35). ROI cropping was performed based on the tumor segmentation mask, and a minimum bounding box was generated according to the tumor contour to remove the cranial background region; On this basis, voxel-wise zero-masking was further applied to background pixels outside the mask to reduce interference from non-tumor tissue on deep feature extraction. Subsequently, the images underwent intensity normalization and size standardization to meet the input specifications of the different networks, preparing them for subsequent analysis and modeling.

### 2.5 Computation of 2D and 3D intratumoral heterogeneity scores

To quantitatively assess tumor-associated heterogeneity in gliomas, this study applied the ITH method developed by Li et al. (22) to map local image intensity features onto topologically structured subregions and performed modeling and analysis at both the 2D and 3D scales. To comprehensively characterize the heterogeneous features of different spatial regions, the relevant metrics were calculated independently for the WT region, the PE region, and the combined tumor-edema region. The PE region was used to characterize microenvironmental heterogeneity associated with tumor infiltration.

2D ITH is used to quantify the spatial heterogeneity of tumors within representative axial slices; its core concept is to reflect the structural complexity of tumors by analyzing the spatial connectivity of different tissue phenotypes. Specifically, the axial slice with the largest tumor area is first selected from the three-dimensional tumor mask as the representative plane. Subsequently, a 2×2 sliding window is used to scan the image pixel by pixel within this 2D region, and basic statistical features—including mean, standard deviation, maximum, and minimum—are extracted from each window to construct a pixel-level feature vector. Based on the feature matrix of all valid pixels, the K-means clustering method is employed for unsupervised classification of pixels, decomposing them into multiple subregions with relatively consistent phenotypes. The number of clusters, K, is adaptively determined using the elbow method based on the sum of squared errors (SSE) to minimize bias resulting from arbitrary parameter settings. After obtaining the cluster label map, for each cluster region *i*, it is further divided into several disconnected connected components, denoted as $n_{i}$, based on 8-neighborhood connectivity, and the area of the largest connected region, $S_{i,\max}$, is calculated. Finally, by performing an area-weighted integration of the spatial distributions of all cluster regions, a two-dimensional heterogeneity score is obtained, defined as follows:


$$2\text{D ITH}=1-\frac{1}{{\text{S}}_{\text{total}}}\sum_{i=1}^{K}\frac{S_{i,\max}}{n_{i}}$$

In this context, ${\text{S}}_{\text{total}}$ represents the total area of the target analysis region on the largest cross-section. This metric reflects the structural complexity of the tumor by integrating the spatial dispersion of different phenotypic regions. The more dispersed the structure and the weaker the spatial connectivity, the higher the ITH score (36, 37).

Building on the 2D analysis, 3D ITH is further extended to the volumetric data level to comprehensively capture the anisotropic textural changes and volumetric distribution characteristics of tumors in three-dimensional space. Across the entire three-dimensional mask, voxel-level feature extraction is performed using a 2×2×2 sliding window, and clustering is conducted based on the K-means algorithm. During the topological analysis stage, to preserve structural continuity in three-dimensional space, 26-neighborhood connectivity (including the connectivity relationships among faces, edges, and vertices) is used to perform a connected component decomposition on each clustered region. This yields the number of connected components ${\text{m}}_{\text{i}}$ for class i and calculates the maximum connected component volume $V_{i,\max}$. The final three-dimensional heterogeneity score is defined as:


$$3\text{D ITH}=1-\frac{1}{{\text{V}}_{\text{total}}}\sum_{i=1}^{K}\frac{V_{i,\max}}{{\text{m}}_{i}}$$

In this context, ${\text{V}}_{\text{total}}$ represents the total volume of the entire three-dimensional analysis region. This metric reflects the complexity and heterogeneity of the tumor’s spatial structure from a 3D perspective and is particularly suitable for describing gliomas with invasive growth characteristics (37, 38).

### 2.6 Feature selection and model construction

In this study, the clinical inclusion variables included sex, age, pathological grade, and IDH mutation status. We first extracted DL, radiomics, and ITH features, removed low-variance components from these feature variables, and performed Z-score standardization. For the DL features, feature matrices were constructed separately for different network architectures and integrated with the clinical variables into the feature space. Subsequently, for the independent feature matrices of DL, Radiomics, and ITH from each architecture, we performed feature selection using a Cox proportional hazards model combined with the Least Absolute Shrinkage and Selection Operator (LASSO), evaluated the models via 10-fold cross-validation, and constructed the LASSO-Cox model; A subset of features with the highest prognostic value was identified. Each model constructs risk models with varying proportions based on the filtered feature subsets and outputs a risk score. Building on this, the hybrid model employs two strategies. First, we concatenate the feature subsets selected from each individual model to form a joint feature matrix, and then perform cross-model joint modeling using Cox regression with an Elastic Net regularization term. This strategy comprehensively covers all possible combinations of two-model (DL+Radiomics, DL+ITH, Radiomics+ITH) and three-model (DL+Radiomics+ITH) configurations, with the models outputting a fused risk score; Second, we constructed a scoring-level Stacking Cox model, using the risk scores output by the single-model Cox models as secondary input features. These were fed into a second-stage LASSO-Cox meta-learner for secondary fitting decisions, thereby achieving in-depth integration of prognostic information from multiple models. Finally, all constructed single-model and fusion models under different architectures were comprehensively evaluated and compared in terms of predictive performance using the Harrell consistency index (C-index) and its 95% confidence interval on both the training and testing sets.

### 2.7 Statistical analysis

Statistical analyses were performed using R (version 4.3.0) and Python (version 3.10). Continuous variables were expressed as mean ± standard deviation (mean ± SD) for normally distributed data and as median (interquartile range) for non-normally distributed data. Comparisons between groups were performed using the independent-samples t-test or the Mann–Whitney U test; categorical variables were expressed as frequencies (percentages) and analyzed using the chi-square (χ²) test or Fisher’s exact test. Model performance was primarily evaluated using the C-index to assess discriminatory ability, and Kaplan–Meier survival analysis combined with the log-rank test was used to compare survival differences among different risk groups. Additionally, time-dependent receiver operating characteristic (ROC) curves were used to evaluate the predictive ability of the optimal fusion model at different time points, with the area under the curve (AUC) used to quantify its discriminatory performance. The calibration performance of the predictive model was evaluated using time-dependent calibration curves, with preset time points at 12, 18, 24, 30, and 36 months. To rigorously assess internal calibration and minimize potential overfitting, we employed a bootstrap resampling method with 1,000 iterations; additionally, we evaluated the model’s potential clinical utility through decision curve analysis (DCA), which quantifies the standardized net benefit across continuous intervals of threshold probabilities. To appropriately handle missing observations, we used the survival analysis extension of DCA. This method incorporates Kaplan-Meier estimates when calculating the actual event rates at specified prediction time points. To evaluate the predictive value of the incremental model, different clinical models were constructed as baselines, and the C-index was compared after incorporating the imaging model as an additional covariate; the 95% CI was estimated using 500 bootstrap repetitions. All tests were two-sided, and a P value < 0.05 was considered statistically significant.

## 3 Results

### 3.1 Baseline characteristics

This study included a total of 292 patients, comprising 183 in the training set and 109 in the test set. The mean age of the overall cohort was 56.9 ± 15.1 years. The difference was statistically significant (P = 0.042). Regarding tumor grade, there was a significant difference between the two groups (P = 0.004). The proportion of high-grade tumors was higher in the training set (90.7%), whereas in the test set, it was 78.0%. In terms of extent of surgical resection, the difference between the two groups was statistically significant (P < 0.001), with a higher proportion of complete resections in the test set (73.4% vs. 50.8%). There was no statistically significant difference in IDH mutation status between the two groups (P = 0.064); both groups were predominantly wild-type. The proportions of fatal events were similar between the two groups. There were statistically significant differences between the two groups in median follow-up time (P < 0.001) and mean overall survival (P < 0.001) in the training and test sets, respectively (**Table 1**).

**Table 1:** Baseline characteristics of patients in the training and test cohorts.

Characteristics	Total cohort	Training cohort	Test cohort	P value
Age (mean ± SD)	56.9 ± 15.1	58.2 ± 15.4	54.6 ± 14.4	0.042
Sex				0.348
Male	167 (57.2%)	109 (59.6%)	58 (53.2%)	
Female	125 (42.8%)	74 (40.4%)	51 (46.8%)	
Grade				0.004
Low	41 (14.0%)	17 (9.3%)	24 (22.0%)	
High	251 (86.0%)	166 (90.7%)	85 (78.0%)	
Extent of resection				<0.001
Gross total resection	173 (59.2%)	93 (50.8%)	80 (73.4%)	
Subtotal resection	91 (31.2%)	63 (34.4%)	28 (25.7%)	
Partial resection	1 (0.3%)	0	1 (0.9%)	
Biopsy	27 (9.2%)	27 (14.8%)	0	
Adjuvant Therapy				/
Chemoradiotherapy	/	/	66 (60.6%)	
Chemotherapy	/	/	21 (19.3%)	
Radiotherapy	/	/	5 (4.6%)	
No treatment	/	/	17 (15.6%)	
IDH mutation				0.064
Wildtype	230 (78.8%)	150 (82.0%)	80 (73.4%)	
Mutated	60 (20.5%)	33 (18.0%)	27 (24.8%)	
Unknown	2 (0.7%)	0	2 (0.7%)	
Survival status				0.729
Death	123 (42.1%)	79 (43.2%)	44 (40.4%)	
Alive	169 (57.9%)	104 (56.8%)	65 (59.6%)	
Median follow-up time (IQR)	12.67(6.25-24.24)	10.49(4.92-18.23)	18.40(10.18-31.04)	<0.001
OS (mean ± SD)	17.7 ± 16.6	14.6 ± 16.0	23.0 ± 16.5	<0.001

### 3.2 Performance of different models

For detailed results on the various individual models and their fusion models, please refer to **Tables 2** and **3**. Within a single DL network architecture, DenseNet121 demonstrated relatively good predictive performance, with C-index values of 0.754 (95% CI: 0.701–0.806) and 0.663 (95% CI: 0.589–0.738) on the training and test sets, respectively. This DL model ultimately retained five key features (feature_522, feature_628, feature_793, Age, IDH); in contrast, the radiomics model retained 4 omics features after screening (original_shape_Sphericity, original_glrlm_LongRunLowGrayLevelEmphasis, wavelet-LHL_ngtdm_Strength, wavelet-HLL_glcm_MCC), and its overall predictive performance was relatively weak, with C-index values of 0.668 (95% CI: 0.600–0.735) and 0.648 (95% CI: 0.563–0.733) for the training and testing sets, respectively; The ITH feature space was derived from six types of MRI images across three regions—the WT region, the PE region, and the combined tumor-edema region—and in both 2D and 3D dimensions, yielding a total of 36 heterogeneity indices. After screening, 11 heterogeneity features were retained, and the ITH model achieved C-indices of 0.712 and 0.663 on the training and testing sets, respectively. In the fusion strategy, the predictive performance of each combined model showed a relative improvement compared to that of the individual models. In the comparison of the two models, the combination that did not include DL features (Rad+ITH) achieved a test set C-index of 0.660 (95% CI: 0.577–0.744); in contrast, the dual-model combinations incorporating DenseNet121 features (DL+Rad and DL+ITH) achieved test set C-indices of 0.688 (95% CI: 0.611–0.765) and 0.689 (95% CI: 0.614–0.763), respectively. Further integration of all three models (densenet121_DL+Rad+ITH) demonstrated relatively better predictive performance. These three models ultimately selected 17 features (including 3 DL features, 3 radiomics features, 9 ITH features, as well as Age and IDH), achieving a training set C-index of 0.801 (95% CI: 0.750–0.853) and a test set C-index of 0.710 (95% CI: 0.638–0.783). Additionally, the corresponding Stacking ensemble model (densenet121_fusion Stacking) maintained a similar level of predictive performance (test set C-index = 0.701). The specific number of features retained by each model after screening is shown in **Supplementary Table 2**.

**Table 2:** Training and testing set C-index values for all single models.

	Training cohort		Test cohort	
Model	C-index	95% CI	C-index	95% CI
Radiomics	0.668	0.600–0.735	0.648	0.563–0.733
ITH	0.712	0.655–0.769	0.663	0.574–0.752
DL_resnet18	0.734	0.676–0.792	0.661	0.583–0.739
DL_resnet34	0.808	0.760–0.855	0.662	0.582–0.742
DL_resnet50	0.776	0.728–0.824	0.616	0.543–0.690
DL_densenet121	0.754	0.701–0.806	0.663	0.589–0.738
InceptionV3	0.774	0.723–0.824	0.635	0.555–0.714

**Table 3:** Training and testing set C-index values for all fusion combinations.

	Training cohort		Test cohort	
Model	C-index	95% CI	C-index	95% CI
resnet18_DL+Rad	0.770	0.715–0.825	0.672	0.587–0.756
resnet18_DL+ITH	0.780	0.727–0.832	0.683	0.601–0.764
resnet18_DL+Rad+ITH	0.796	0.743–0.848	0.691	0.610–0.771
resnet18_fusion Stacking	0.771	0.716–0.827	0.680	0.601–0.759
resnet34_DL+Rad	0.830	0.784–0.875	0.672	0.590–0.755
resnet34_DL+ITH	0.830	0.784–0.876	0.659	0.575–0.743
resnet34_DL+Rad+ITH	0.842	0.798–0.886	0.675	0.592–0.758
resnet34_fusion Stacking	0.822	0.775–0.869	0.683	0.603–0.763
resnet50_DL+Rad	0.800	0.753–0.846	0.637	0.558–0.716
resnet50_DL+ITH	0.807	0.761–0.852	0.656	0.579–0.733
resnet50_DL+Rad+ITH	0.821	0.776–0.867	0.653	0.577–0.730
resnet50_fusion Stacking	0.798	0.752–0.845	0.654	0.580–0.729
densenet121_DL+Rad	0.780	0.727–0.833	0.688	0.611–0.765
densenet121_DL+ITH	0.784	0.733–0.835	0.689	0.614–0.763
densenet121_DL+Rad+ITH	0.801	0.750–0.853	0.710	0.638–0.783
densenet121_fusion Stacking	0.784	0.730–0.838	0.701	0.630–0.772
inception_v3_DL+Rad	0.800	0.751–0.848	0.669	0.591–0.747
inception_v3_DL+ITH	0.800	0.752–0.848	0.671	0.591–0.751
inception_v3_DL+Rad+ITH	0.811	0.763–0.858	0.682	0.605–0.758
inception_v3_fusion Stacking	0.797	0.748–0.847	0.674	0.594–0.755
Rad+ITH	0.732	0.673–0.791	0.660	0.577–0.744
Rad+ITH_Stacking	0.723	0.663–0.784	0.675	0.587–0.762

### 3.3 Prognostic analysis of the optimal model

We used the median risk score from the training set (cutoff = 0.234) as the stratification threshold. The training set included 92 patients in the low-risk group and 91 in the high-risk group; the high-risk group had a higher risk of death than the low-risk group (HR = 4.375, 95% CI: 2.662–7.191, P < 0.001); In the test set (39 patients in the low-risk group and 70 in the high-risk group), an increased risk of mortality was also observed in the high-risk group (HR = 3.555, 95% CI: 1.887–6.697, P < 0.001). As shown in **Figure 2**, the Kaplan–Meier analysis revealed a clear divergence in the survival curves between the two groups, indicating that the model possesses good risk stratification capability. We recorded the number of patients at risk at each time point (0, 6, 12, 18, 24, 30, and 36 months): in the training set, the number of patients at risk in the low-risk and high-risk groups at each time point was 92, 77, 55, 37, 26, 17, and 12, and 91, 52, 26, 10, 4, 3, and 2, respectively; in the test set, the corresponding numbers were 39, 36, 34, 26, 24, 19, and 15 for the low-risk group, and 70, 59, 42, 29, 20, 13, and 8 for the high-risk group. Time-dependent ROC analysis showed (**Figure 3**) that in the training set, the AUC values of the hybrid model at 12, 18, 24, 30, and 36 months were 0.859, 0.913, 0.927, 0.956, and 0.945, respectively. In the test set, the corresponding AUCs were 0.705, 0.721, 0.746, 0.800, and 0.873, indicating that the model exhibits good stability and generalizability in medium- to long-term survival prediction. Calibration curves show that the model’s predicted values align well with observed values at most time points (**Figure 4**). Based on the Cox model framework, we estimated the observed probabilities at each time point using the Kaplan-Meier method and compared them with the model’s predicted probabilities. Overall, the predicted values at each time point corresponded well with the observed values; although the relatively small sample size resulted in some variability in the observed probabilities at each time point, this suggests that the model possesses robust calibration capabilities. In addition, we used DCA to evaluate the model’s clinical utility within a high-risk threshold range of 0.05 to 0.40 (corresponding to a benefit-risk ratio of approximately 1:15 to 2:3) (**Figure 5**). The DCA showed that, based on the five time points of 12, 18, 24, 30, and 36 months, with the high-risk threshold range set at 0.05 to 0.40, net benefit was expressed as standardized net benefit. In the training set, the model demonstrated good positive net benefit at all time points; in the test set, the medium- to long-term predictions at 24, 30, and 36 months also maintained good positive net benefits across the entire broad threshold range; however, the short-term predictions at 12 and 18 months showed a decline in net benefits under higher risk thresholds. Overall, this fusion model demonstrated relatively good and stable potential clinical utility in medium- to long-term clinical applications spanning 24 to 36 months.

**Figure 2:**
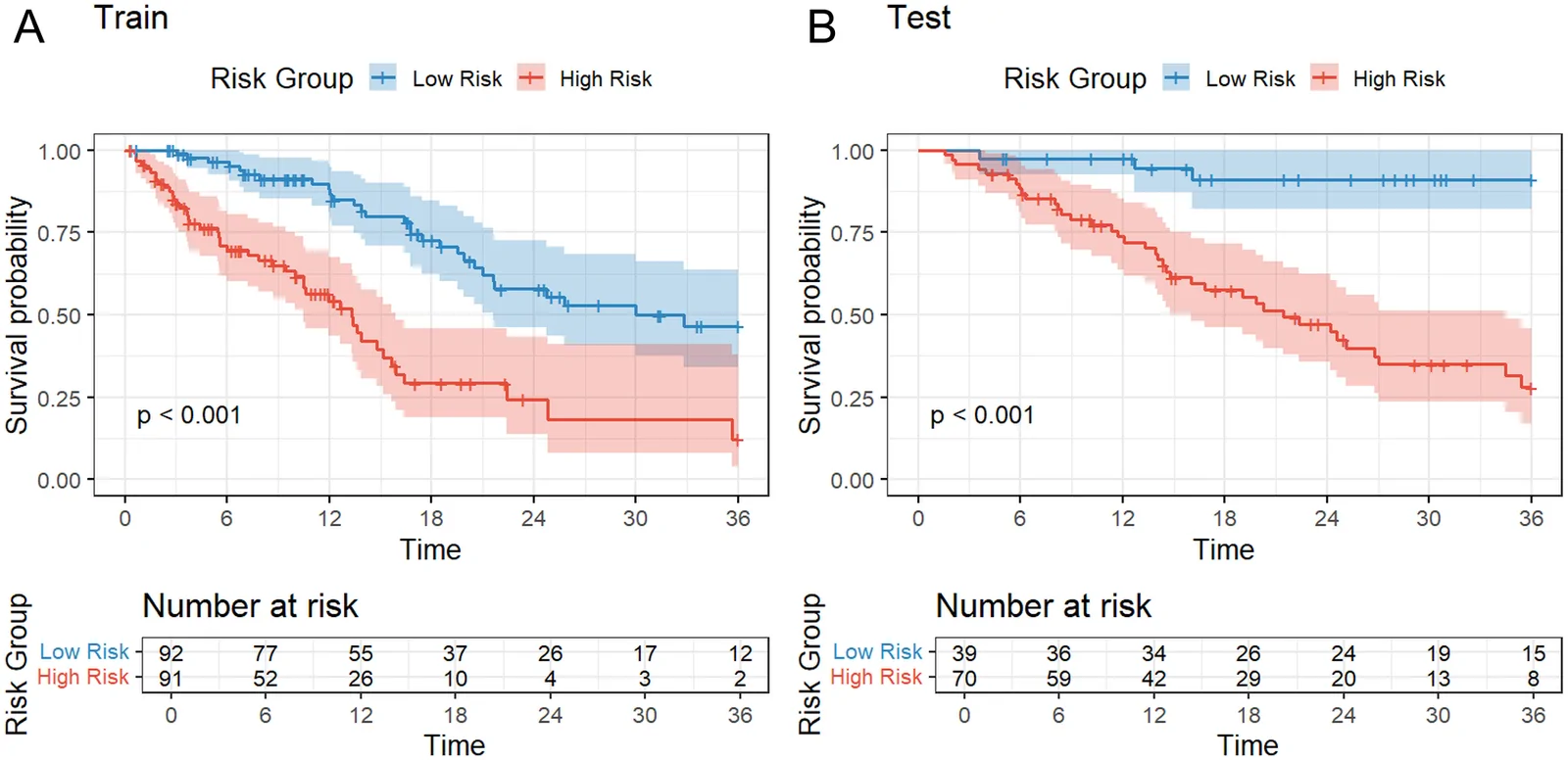
The Kaplan-Meier survival curves of the patients in the training cohorts **(A)** and the test cohorts **(B)** are displayed as low-risk (blue) and high-risk (red) groups. The OS differences between the low- and high-risk groups were assessed by log-rank tests.

**Figure 3:**
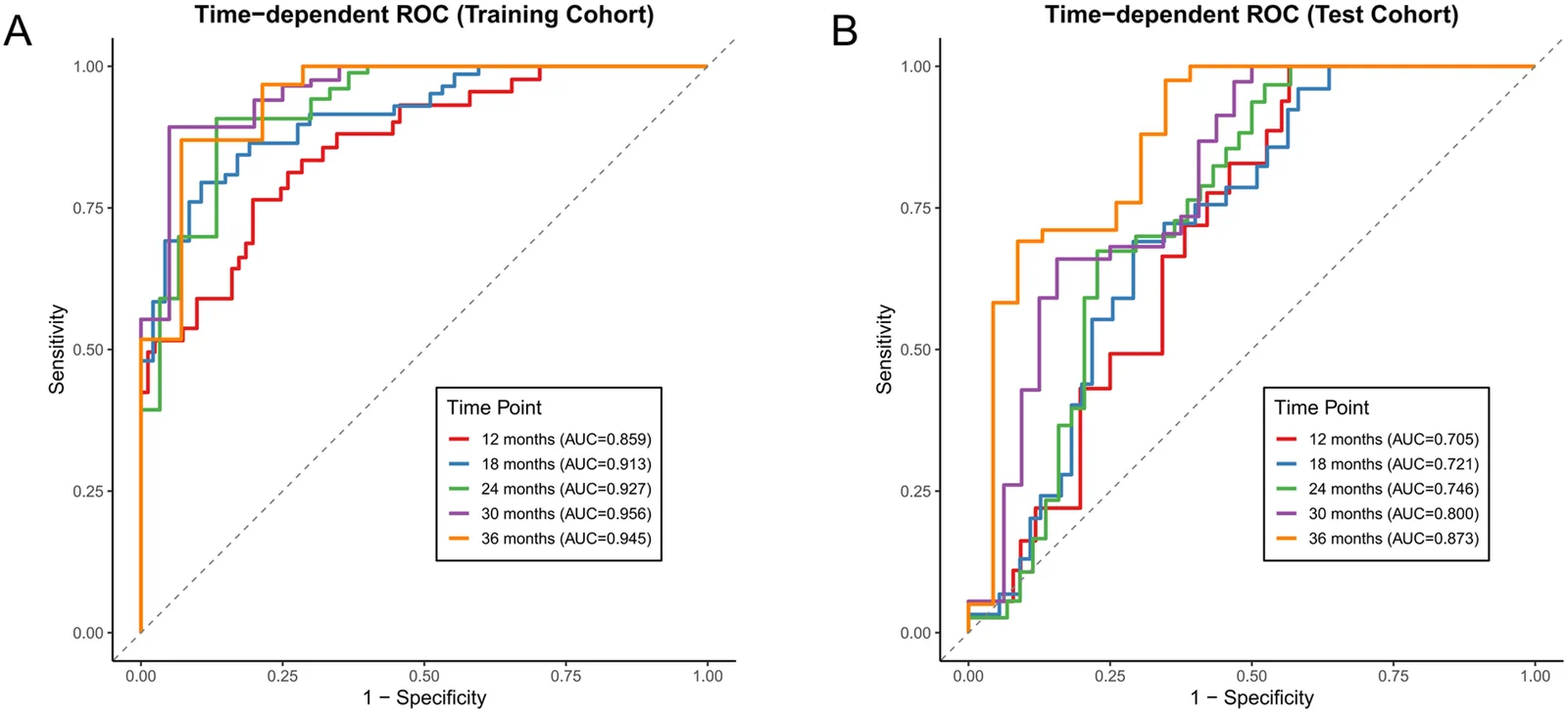
ROC curves for time-dependent predictions at 12, 18, 24, 30, and 36 months in the training cohorts **(A)** and testing cohorts **(B)**.

**Figure 4:**
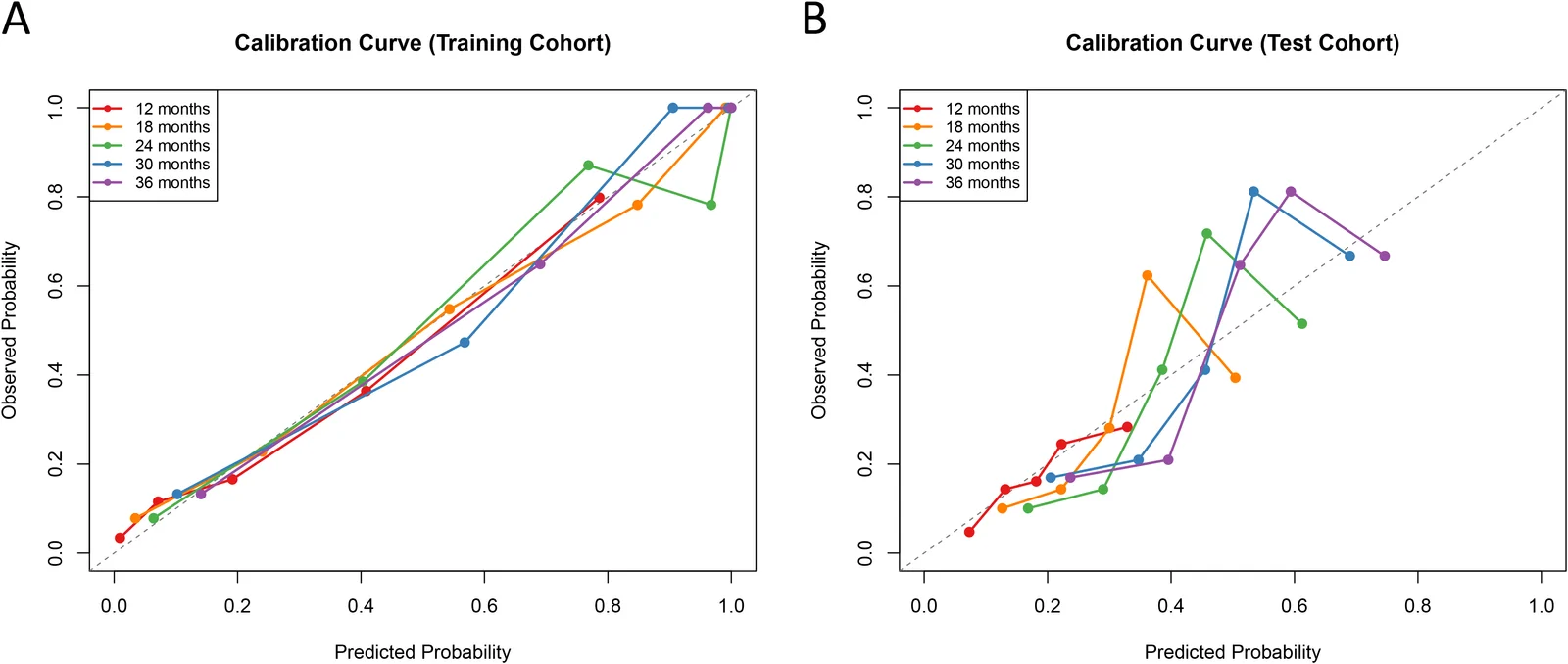
Calibration curves for time-dependent predictions at 12, 18, 24, 30, and 36 months in the training cohorts **(A)** and testing cohorts **(B)**.

**Figure 5:**
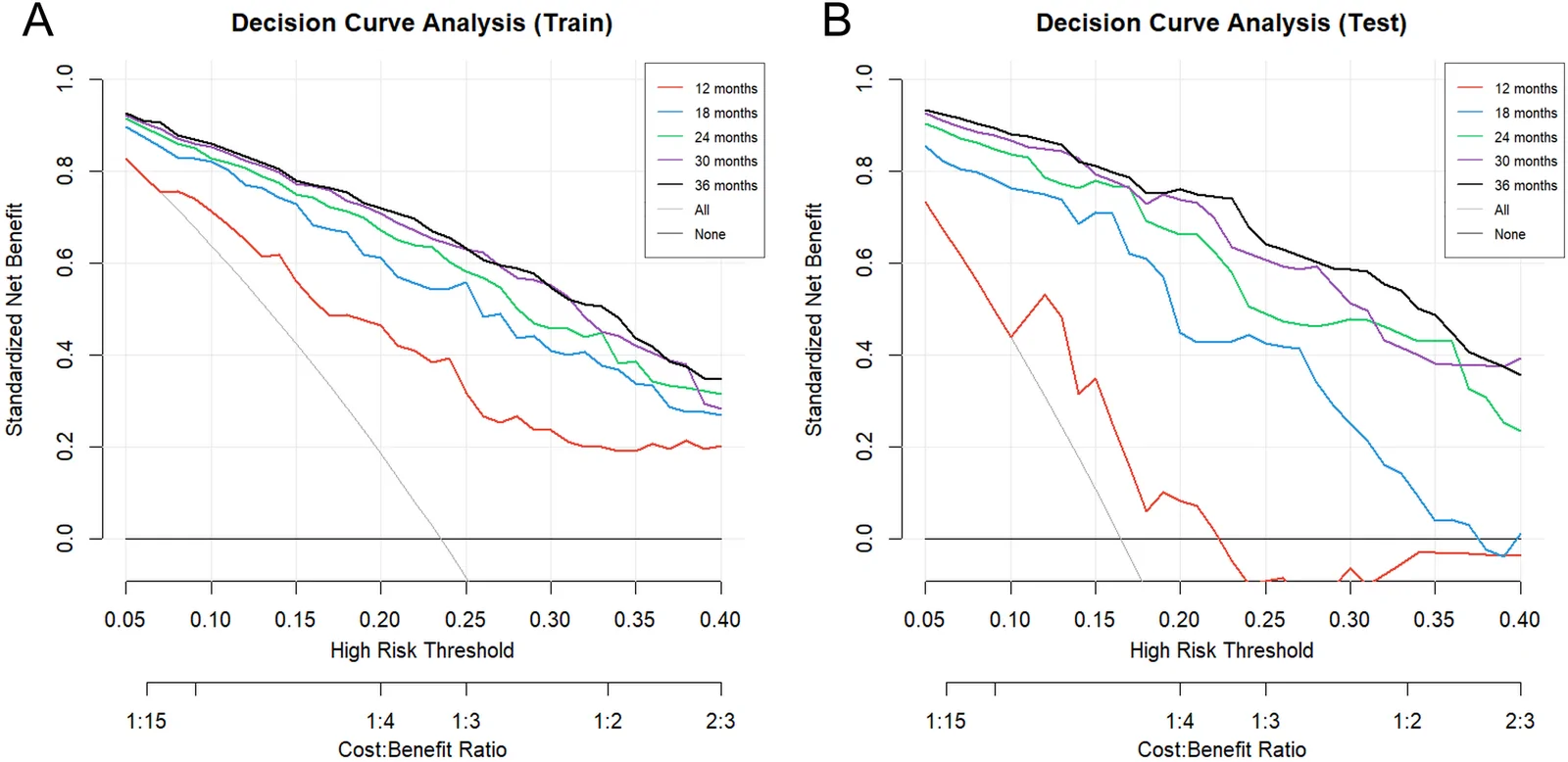
Decision curve analysis (DCA) for time-dependent predictions at 12, 18, 24, 30, and 36 months in the training cohorts **(A)** and testing cohorts **(B)**.

### 3.4 Incremental predictive value of hybrid models

The optimal hybrid model demonstrated varying degrees of incremental discriminatory power compared to each baseline clinical model (**Table 4**). In the training set, compared with the Grade, IDH, Grade+IDH, and full clinical models, the ΔC-index after adding the risk score was +0.267 (95% CI: 0.212–0.325), +0.204 (95% CI: 0.150–0.262), +0.194 (95% CI: 0.141–0.248), and +0.080 (95% CI: 0.034–0.142), respectively. All comparisons were statistically significant (P < 0.001 for all), suggesting that the risk score provides an independent, incremental predictive contribution after fully adjusting for known clinical factors. In the test set, compared with the baseline models—Grade, IDH, Age + IDH, and Grade + IDH—the ΔC-index of the fusion model increased by +0.091 (P = 0.004), +0.093 (P = 0.010), +0.023 (P = 0.030), and +0.055 (P = 0.048), respectively, with all increases being statistically significant. Based on the comprehensive clinical model incorporating Grade, IDH, Age, and Sex, the ΔC-index was +0.022 (95% CI: −0.004–0.074, P = 0.126); the increase was relatively limited and did not reach statistical significance; This result reflects, to some extent, that when the baseline model already incorporates relatively comprehensive clinical covariates, the marginal contribution of the risk score tends to narrow; its actual clinical significance requires further validation in larger-scale cohorts. In summary, this integrated model can, to a certain extent, capture prognostic information that traditional clinical variables fail to fully reflect and may have potential clinical added value.

**Table 4:** The incremental value of mixed models compared to clinical models.

Cohort	Clinical model	Clinical model C-index (95% CI)	Fusion model C-index (95% CI)	ΔC-index (95% CI)	P value
Training	Grade	0.531 (0.499–0.563)	0.798 (0.739–0.850)	+0.267 (0.212–0.325)	<0.001
	IDH	0.595 (0.557–0.631)	0.799 (0.740–0.818)	+0.204 (0.150–0.262)	<0.001
	Age + IDH	0.708 (0.612–0.767)	0.801 (0.750–0.853)	+0.093 (0.041–0.164)	<0.001
	Grade + IDH	0.605 (0.571–0.645)	0.799 (0.741–0.849)	+0.194 (0.141–0.248)	<0.001
	Grade + IDH + Age + Sex	0.717 (0.656–0.776)	0.797 (0.744–0.854)	+0.080 (0.034–0.142)	<0.001
Testing	Grade	0.607 (0.522–0.693)	0. 698 (0.613–0.784)	+0.091 (0.020–0.155)	0.004
	IDH	0.606 (0.551–0.663)	0.699 (0.622–0.784)	+0.093 (0.033–0.167)	0.010
	Age + IDH	0.687 (0.607–0.771)	0.710 (0.638–0.783)	+0.023 (0.001–0.076)	0.030
	Grade + IDH	0.650 (0.585–0.718)	0.705 (0.632–0.790)	+0.055 (0.003–0.121)	0.048
	Grade + IDH + Age + Sex	0.687 (0.636–0.791)	0.709 (0.658–0.809)	+0.022 (-0.004–0.074)	0.126

## 4 Discussion

Based on clinical characteristics and multi-parameter MRI data, this study developed a survival prediction model that integrates DL, radiomics, and ITH features, while systematically comparing different combinations of these features. The results further demonstrate that the integration of multi-source information holds significant value in the prognostic assessment of gliomas. In previous studies, methods for predicting glioma prognosis have gradually shifted from traditional radiological analysis to artificial intelligence-driven models (7, 39–42). Data-driven approaches can uncover latent patterns within high-dimensional imaging information, thereby providing a more objective and quantitative basis for prognostic assessment. Existing studies have shown that a histogram-based model constructed using T2WI radiomic features achieved C-indices of 0.83 and 0.75 in the training and validation sets, respectively (39), confirming its potential for personalized prognostic assessment. However, traditional studies have largely relied on single image sequences and have failed to fully utilize multidimensional information and spatial structural features. Given the significant spatial heterogeneity within gliomas in terms of cell density, angiogenesis, and infiltration characteristics (43–45), unidimensional features often fail to comprehensively characterize the complex biological behavior of tumors. For this reason, strategies for fusing multi-source images and various features have attracted significant attention in recent years (46). By integrating cross-dimensional information to achieve complementary advantages, this strategy effectively overcomes the limitations of single-method approaches, thereby further enhancing the stability and accuracy of predictive models. This trend underscores the core objective of current research: while maintaining model interpretability, there is an urgent need to explore more complementary combinations of multi-source features to precisely enable personalized prognostic assessment of gliomas. Preliminary explorations have been conducted in this area; for example, Marasi et al. (41) extracted latent spatial features from MRI using a pre-trained 3D network and combined them with clinical information. By employing random forests or multilayer perceptrons for modeling, they achieved moderate predictive performance in a multicenter cohort. This study affirmed the value of DL features in risk stratification and highlighted the advantages of multi-source information fusion. To further overcome existing limitations, this study developed a more comprehensive integration framework: at the feature level, it deeply integrated DL features pre-trained on ImageNet with clinical variables, radiomic features, and ITH scores, thereby enabling multidimensional tumor characterization ranging from higher-order semantics and microscopic texture to macroscopic spatial topology. Furthermore, this study systematically compared single and hybrid models within a unified analytical framework, quantified the specific contributions of different feature types, and relatively improved the accuracy of survival prediction.

The results of this study show that, under most architectures, the predictive performance of multi-feature fusion models is generally no worse than that of the corresponding single-feature baseline models; however, the performance differences across modalities are relatively limited, which may indicate a certain degree of information overlap among different feature types. The triple-fusion model (densenet121_DL+Rad+ITH) achieved a C-index of 0.710 on the test set, representing an improvement over the dual-fusion combinations (densenet121_DL+ITH = 0.689, densenet121_DL+Rad = 0.688), though the improvement was modest. When considered in isolation, the predictive performance of radiomics models is relatively limited, a fact closely related to their inherent limitations in capturing higher-order spatial structures and cross-scale features—while relying solely on radiomics features can describe local texture and grayscale distribution to a certain extent, it remains inadequate for characterizing complex nonlinear image patterns (47). From the perspective of fusion models, the combination of DL features and ITH features yields a relatively consistent performance improvement in most architectures, suggesting that the two are complementary at the information level. In terms of the information contribution mechanisms of each model, DL features extract high-dimensional semantic information and latent structural patterns through pre-trained networks, capturing complex nonlinear image relationships without the need for explicit feature engineering (48, 49), while ITH scores quantify the degree of heterogeneity among different subregions within a tumor from a spatial distribution perspective, further reflecting the spatial patterns of tumor growth, invasion extent, and microenvironment composition (50, 51). In contrast, clinical features provide key information at the individual patient level, reflecting the overall disease progression and treatment history (52), thereby compensating to some extent for the macroscopic biological behaviors that imaging features cannot directly express. They form a complementary relationship at the information level; by integrating representations from different dimensions, they help reduce the bias introduced by a single feature source, thereby constructing relatively more stable predictive models and enhancing model performance and stability. However, it should be noted that hybrid models do not always yield consistent results. Our single DL model (DL_resnet34) achieved C-indices of 0.808 and 0.662 on the training and test sets, respectively; Upon incorporating ITH features (resnet34_DL+ITH), although its fitting performance on the training set further improved to 0.830, its performance on the independent test set declined slightly (0.659). This phenomenon indicates that feature complementarity is just as important as the model’s generalization ability. At the same time, an incremental value analysis further revealed the independent prognostic contribution of the fusion risk score relative to clinical variables. The risk score demonstrated the most significant incremental benefit when compared to a simple baseline model containing only a single clinical indicator (Grade or IDH), indicating that imaging-driven models can effectively capture prognostic information not covered by individual molecular or pathological markers. However, it is worth noting that when the baseline clinical model already incorporated a relatively comprehensive set of clinical covariates (Grade + IDH + Age + Sex), the incremental ΔC-index of the risk score in the test set narrowed to +0.022 and did not reach statistical significance (P = 0.126). This result somewhat limits the marginal contribution of the imaging model, and its practical clinical significance requires further validation in larger, multicenter, independent cohorts. Therefore, when constructing combined prognostic models for gliomas, one should adopt a task-oriented feature selection strategy. By carefully selecting combinations of features that are highly complementary and have low redundancy, one can strike the optimal balance between complex representational capacity and stringent generalization constraints, rather than simply stacking all available features (53).

Of course, this study has several limitations. The study employed a retrospective design, which may introduce selection bias and limit the ability to draw causal inferences. Future prospective studies are needed to validate these findings and provide a reference for risk stratification, treatment planning, and patient communication. Furthermore, since the imaging data were obtained from different scanning devices and parameter settings, variations in imaging resolution and protocols may affect the stability of features, which in turn may reduce the model’s reproducibility and robustness across different clinical settings; image segmentation relies on manual annotation, which may introduce inter-observer variability, future studies could assess the impact of automated segmentation algorithms on predictive results; furthermore, this study did not include key molecular biological markers such as O^6^-methylguanine-DNA methyltransferase (MGMT) promoter methylation status, chromosome 1p and 19q (1p/19q) codeletion, and telomerase reverse transcriptase (TERT) status, which to some extent limits the comprehensive characterization of tumor biological features. In the future, a multi-omics model could be developed by integrating radiomics, genomics, and comprehensive pathological information to further optimize risk stratification capabilities. Subsequent studies should conduct validation using larger, multicenter datasets, with a particular focus on systematically evaluating the model’s generalizability under different imaging protocols and equipment conditions to facilitate its translation into clinical practice.

## 5 Conclusion

This study explored the feasibility of predictive models based on clinical features and multiparametric MRI features for assessing survival in gliomas. The analysis showed that a hybrid model integrating clinical information, DL, radiomics, and ITH offers certain advantages in overcoming the limitations of single-source data. It maintains relatively good predictive performance in both the training and testing cohorts, demonstrating potential value for prognostic assessment. However, the fusion model is still in the exploratory stage, and its generalizability and actual clinical net benefit require validation through rigorous prospective studies in the future.

## Data Availability

The raw data supporting the conclusions of this article will be made available by the authors, without undue reservation.
