# Supplementary material for: An integrated prognostic model incorporating clinical characteristics, multiparametric MRI radiomics, deep learning features, and intratumoral heterogeneity for glioma patients
Source: Front Oncol. 2026 Sep 21;16:1873715. doi: 10.3389/fonc.2026.1873715 (PMC13635987; doi:10.3389/fonc.2026.1873715)
Supplement: Supplementary file 1 [file Table1.docx]

**Supplementary Table 1.** Scan protocol parameters.

| **Scanner** | **Field strength** | **Sequence** | **TR (ms)** | **TE (ms)** | **Thickness (mm)** | **FOV (mm²)** | **Matrix** |
| --- | --- | --- | --- | --- | --- | --- | --- |
| United Imaging  uMR 670 | 1.5T | T1WI | 2165 | 11.86 | 5.0 | 230 × 200 | 256 × 218 |
|  |  | T2WI | 5236 | 107.4 | 5.0 | 230 × 200 | 320 × 320 |
|  |  | T2-FLAIR | 6173 | 121.68 | 5.0 | 230 × 200 | 256 × 218 |
|  |  | T1CE | 6.18 | 2.78 | 1.5 | 220 × 220 | 256 × 256 |
|  |  | DWI | 4485 | 86 | 5.0 | 230 × 230 | 128 × 128 |
| Siemens  Skyra MR | 3T | T1WI | 200 | 2.49 | 5.0 | 240 × 202 | 300 × 253 |
|  |  | T2WI | 4540 | 95 | 5.0 | 240 × 202 | 600 × 506 |
|  |  | T2-FLAIR | 8000 | 92 | 5.0 | 240 × 202 | 256 × 256 |
|  |  | T1CE | 5.33 | 2.46 | 1.0 | 250 × 250 | 312 × 312 |
|  |  | DWI | 5000 | 96 | 5.0 | 240 × 240 | 130 × 130 |
| GE Healthcare |  | T1WI | 6 | 2.3 | 1.0 | 256 × 256 | 256× 256 |
|  | 3T | T2WI | 2200 | 100 | 1.2 | 256 × 256 | 256× 256 |
|  |  | T2-FLAIR | 5700 | 115 | 1.2 | 256 × 256 | 256× 256 |
|  |  | CE-T1WI | 6 | 2.3 | 1.0 | 256 × 256 | 256× 256 |
|  |  | DWI | 10000 | 99 | 2.0 | 230 × 230 | 256× 256 |

**Supplementary Table 2.** Number of Filtering Features.

| Model |  | Number of Features | Retained Features |
| --- | --- | --- | --- |
| Radiomics |  | 4 | original_shape_Sphericity original_glrlm_LongRunLowGrayLevelEmphasis wavelet-LHL_ngtdm_Strength wavelet-HLL_glcm_MCC |
| ITH |  | 11 | ADC_edema_2D T1C_edema_2D T1C_tumor_2D T1C_whole_2D T1_edema_2D T1_tumor_2D ADC_edema_3D ADC_tumor_3D ADC_whole_3D T1_edema_3D T2_edema_3D |
| DL_resnet18 |  | 4 | feature_22 Age Grade IDH |
| resnet18_DL+Rad |  | 8 | feature_22 Age Grade IDH original_shape_Sphericity original_glrlm_LongRunLowGrayLevelEmphasis wavelet-LHL_ngtdm_Strength wavelet-HLL_glcm_MCC |
| resnet18_DL+ITH |  | 11 | feature_22 Age Grade IDH ADC_edema_2D T1C_whole_2D T1_edema_2D T1_tumor_2D ADC_tumor_3D ADC_whole_3D T2_edema_3D |
| resnet18_DL+Rad+ITH |  | 14 | feature_22 Age Grade IDH original_shape_Sphericity original_glrlm_LongRunLowGrayLevelEmphasis wavelet-LHL_ngtdm_Strength wavelet-HLL_glcm_MCC T1C_tumor_2D T1C_whole_2D T1_tumor_2D ADC_tumor_3D ADC_whole_3D T2_edema_3D |
| resnet18_fusion Stacking |  | 3 | DL Rad ITH |
| DL_resnet34 |  | 20 | feature_18 feature_36 feature_143 feature_152 feature_155 feature_157 feature_161 feature_179 feature_293 feature_299 feature_322 feature_328 feature_349 feature_389 feature_475 feature_476 Sex Age Grade IDH |
| resnet34_DL+Rad |  | 23 | feature_18 feature_36 feature_143 feature_152 feature_155 feature_157 feature_161 feature_179 feature_293 feature_299 feature_322 feature_328 feature_349 feature_389 feature_475 Sex Age Grade IDH original_shape_Sphericity original_glrlm_LongRunLowGrayLevelEmphasis wavelet-LHL_ngtdm_Strength wavelet-HLL_glcm_MCC |
| resnet34_DL+ITH |  | 28 | feature_18 feature_36 feature_143 feature_152 feature_155 feature_157 feature_161 feature_179 feature_293 feature_299 feature_322 feature_328 feature_349 feature_389 feature_475 feature_476 Sex Age Grade IDH ADC_edema_2D T1C_tumor_2D T1C_whole_2D T1_edema_2D T1_tumor_2D ADC_tumor_3D ADC_whole_3D T2_edema_3D |
| resnet34_DL+Rad+ITH |  | 31 | feature_18 feature_36 feature_143 feature_152 feature_155 feature_157 feature_161 feature_179 feature_293 feature_299 feature_322 feature_328 feature_349 feature_389 feature_475 Sex Age Grade IDH original_shape_Sphericity original_glrlm_LongRunLowGrayLevelEmphasis wavelet-LHL_ngtdm_Strength wavelet-HLL_glcm_MCC ADC_edema_2D T1C_tumor_2D T1C_whole_2D T1_edema_2D T1_tumor_2D ADC_tumor_3D ADC_whole_3D T2_edema_3D |
| resnet34_fusion Stacking |  | 3 | DL Rad ITH |
| DL_resnet50 |  | 7 | feature_137 feature_1056 feature_1529 feature_1870 Age Grade IDH |
| resnet50_DL+Rad |  | 11 | feature_137 feature_1056 feature_1529 feature_1870 Age Grade IDH original_shape_Sphericity original_glrlm_LongRunLowGrayLevelEmphasis wavelet-LHL_ngtdm_Strength wavelet-HLL_glcm_MCC |
| resnet50_DL+ITH |  | 16 | feature_137 feature_1056 feature_1529 feature_1870 Age Grade IDH ADC_edema_2D T1C_edema_2D T1C_tumor_2D T1C_whole_2D T1_edema_2D T1_tumor_2D ADC_tumor_3D ADC_whole_3D T2_edema_3D |
| resnet50_DL+Rad+ITH |  | 16 | feature_137 feature_1056 feature_1529 feature_1870 Age Grade IDH original_glrlm_LongRunLowGrayLevelEmphasis wavelet-LHL_ngtdm_Strength wavelet-HLL_glcm_MCC T1C_tumor_2D T1C_whole_2D T1_tumor_2D ADC_tumor_3D ADC_whole_3D T2_edema_3D |
| resnet50_fusion Stacking |  | 3 | DL Rad ITH |
| DL_densenet121 |  | 5 | feature_522 feature_628 feature_793 Age IDH |
| densenet121_DL+Rad |  | 8 | feature_522 feature_628 feature_793 Age IDH original_glrlm_LongRunLowGrayLevelEmphasis wavelet-LHL_ngtdm_Strength wavelet-HLL_glcm_MCC |
| densenet121_DL+ITH |  | 14 | feature_522 feature_628 feature_793 Age IDH ADC_edema_2D T1C_edema_2D T1C_tumor_2D T1C_whole_2D T1_edema_2D T1_tumor_2D ADC_tumor_3D ADC_whole_3D T2_edema_3D |
| densenet121_DL+Rad+ITH |  | 17 | feature_522 feature_628 feature_793 Age IDH original_glrlm_LongRunLowGrayLevelEmphasis wavelet-LHL_ngtdm_Strength wavelet-HLL_glcm_MCC T1C_tumor_2D T1C_whole_2D T1_edema_2D T1_tumor_2D ADC_edema_3D ADC_tumor_3D ADC_whole_3D T1_edema_3D T2_edema_3D |
| densenet121_fusion Stacking |  | 3 | DL Rad ITH |
| DL_inception_v3 |  | 6 | feature_1455 feature_1457 feature_1645 feature_1997 Age IDH |
| inception_v3_DL+Rad |  | 10 | feature_1455 feature_1457 feature_1645 feature_1997 Age IDH original_shape_Sphericity original_glrlm_LongRunLowGrayLevelEmphasis wavelet-LHL_ngtdm_Strength wavelet-HLL_glcm_MCC |
| inception_v3_DL+ITH |  | 14 | feature_1455 feature_1457 feature_1645 feature_1997 Age IDH ADC_edema_2D T1C_tumor_2D T1C_whole_2D T1_edema_2D T1_tumor_2D ADC_tumor_3D ADC_whole_3D T2_edema_3D |
| inception_v3_DL+Rad+ITH |  | 17 | feature_1455 feature_1457 feature_1645 feature_1997 Age IDH original_shape_Sphericity original_glrlm_LongRunLowGrayLevelEmphasis wavelet-LHL_ngtdm_Strength wavelet-HLL_glcm_MCC T1C_tumor_2D T1C_whole_2D T1_tumor_2D ADC_edema_3D ADC_tumor_3D ADC_whole_3D T2_edema_3D |
| inception_v3_fusion Stacking |  | 3 | DL Rad ITH |
| Rad+ITH_Fusion |  | 13 | original_shape_Sphericity original_glrlm_LongRunLowGrayLevelEmphasis wavelet-LHL_ngtdm_Strength wavelet-HLL_glcm_MCC T1C_edema_2D T1C_tumor_2D T1C_whole_2D T1_edema_2D T1_tumor_2D ADC_edema_3D ADC_whole_3D T1_edema_3D T2_edema_3D |
| Rad+ITH_fusion Stacking |  | 2 | Rad ITH |
